# Evaluating the Arteriotomy Size of a New Sutureless Coronary Anastomosis Using a Finite Volume Approach

**DOI:** 10.1007/s12265-023-10367-9

**Published:** 2023-03-21

**Authors:** Hanneke Crielaard, Marieke Hoogewerf, Bart P. van Putte, Frans N. van de Vosse, Georgios J. Vlachojannis, David Stecher, Marco Stijnen, Pieter A. Doevendans

**Affiliations:** 1 LifeTec Group, Eindhoven, The Netherlands; 2 Department of Cardiovascular Biomechanics, University of Eindhoven, Eindhoven, The Netherlands; 3 Department of Biomedical Engineering, Erasmus Medical Center, Rotterdam, The Netherlands; 4 Department of Cardiology, University Medical Center Utrecht, Heidelberglaan 100, 3584CX Utrecht, The Netherlands; 5 Department of Cardiothoracic Surgery, St. Antonius Hospital, Nieuwegein, The Netherlands; 6 Department of Cardiothoracic Surgery, Amsterdam University Medical Center, Amsterdam, The Netherlands; 7 Department of Cardiothoracic Surgery, University Medical Center Utrecht, Utrecht, The Netherlands; 8 Netherlands Heart Institute, Utrecht, The Netherlands

**Keywords:** Coronary artery bypass grafting, Sutureless coronary anastomoses, Computational fluid dynamics, Fractional flow reserve

## Abstract

**Objectives:**

The ELANA® Heart Bypass creates a standardized sutureless anastomosis. Hereby, we investigate the influence of arteriotomy and graft size on coronary hemodynamics.

**Methods:**

A computational fluid dynamics (CFD) model was developed. Arteriotomy size (standard 1.43 mm^2^; varied 0.94 – 3.6 mm^2^) and graft diameter (standard 2.5 mm; varied 1.5 – 5.0 mm) were independent parameters. Outcome parameters were coronary pressure and flow, and fractional flow reserve (FFR).

**Results:**

The current size ELANA (arteriotomy 1.43 mm^2^) presented an estimated FFR 0.65 (39 mL/min). Enlarging arteriotomy increased FFR, coronary pressure, and flow. All reached a maximum once the arteriotomy (2.80 mm^2^) surpassed the coronary cross-sectional area (2.69 mm^2^, i.e. 1.85 mm diameter), presenting an estimated FFR 0.75 (46 mL/min). Increasing graft diameter was positively related to FFR, coronary pressure, and flow.

**Conclusion:**

The ratio between the required minimal coronary diameter for application and the ELANA arteriotomy size effectuates a pressure drop that could be clinically relevant. Additional research and eventual lengthening of the anastomosis is advised.

**Graphical abstract:**

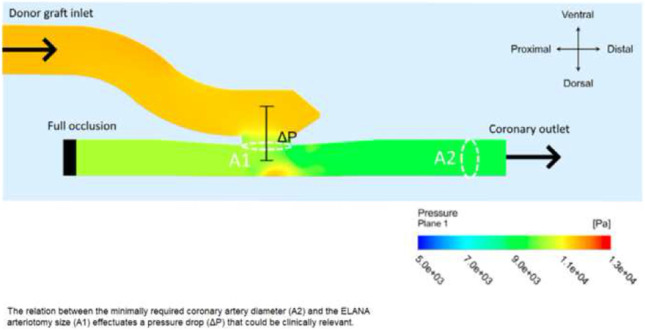

**Supplementary Information:**

The online version contains supplementary material available at 10.1007/s12265-023-10367-9.

## Introduction

Minimally invasive coronary artery bypass grafting (CABG) procedures are still challenging due to complicated anastomotic suturing under limited exposure. A variety of sutureless anastomotic devices were developed to facilitate these procedures, of which the C-Port® and U-clip were most promising. Yet, none of the devices is currently on the market, all for different reasons like device handling or costs [1, 2]. The ELANA® Heart Bypass was recently redesigned for coronary bypass application, following application of the ELANA technique in neurovascular surgery until the uprise of percutaneous stenting [3–7]. The ELANA Heart Bypass differs from other coronary anastomotic techniques since it connects graft to coronary artery by a titanium clip and creates an arteriotomy by excimer (contact) laser thereafter. This technique limits manipulation and trauma, standardizes the procedure, and ensures a proper fit between device and arteriotomy. In contrast to most other techniques, however, the arteriotomy size depends on the device’s size rather than the diameter of the receiving coronary artery.

**Fig. 1:**
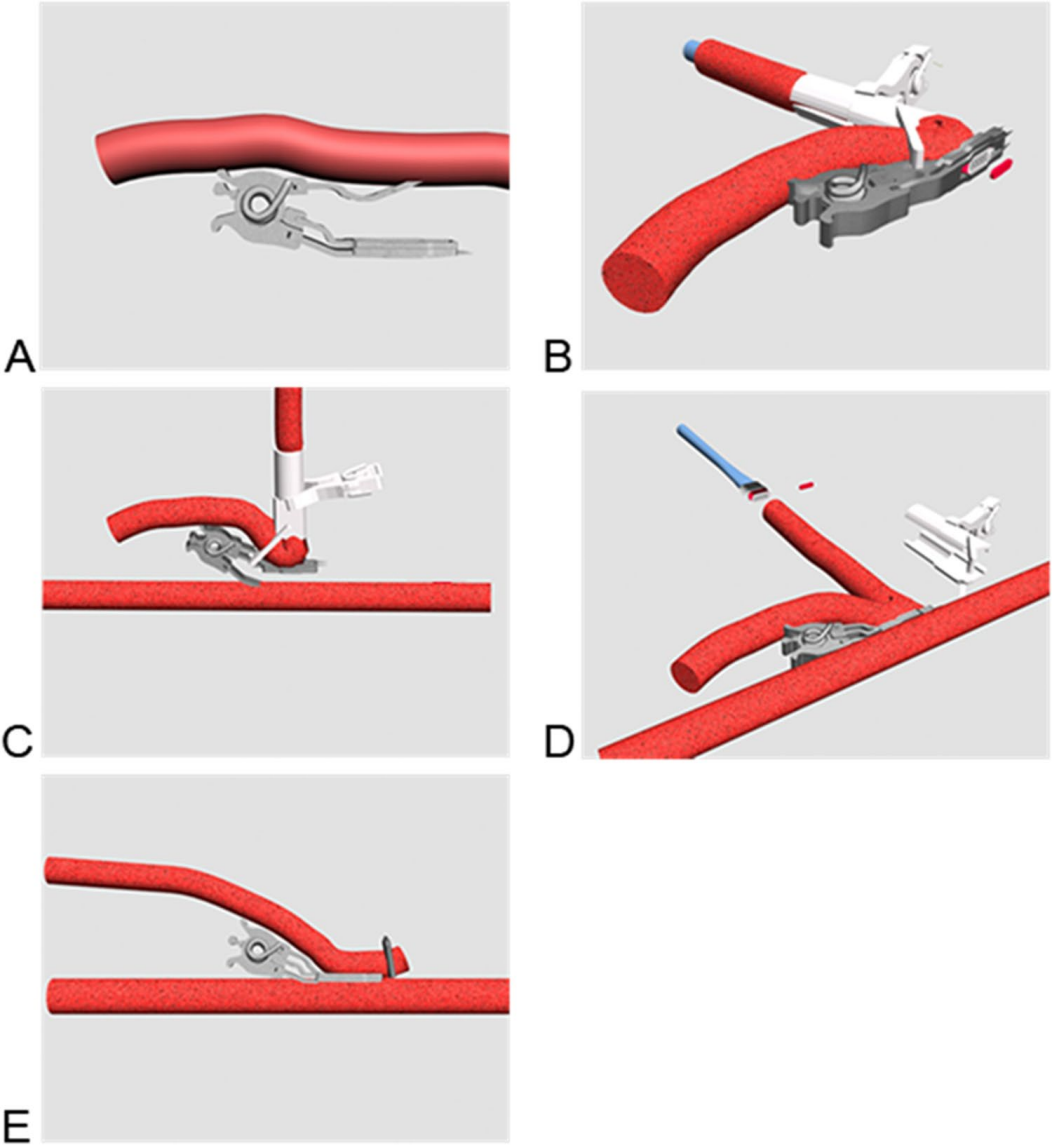
The ELANA Heart Bypass technique. The upper fork of the ELANA Heart Clip is inserted into the lumen of the graft (**a**). The ELANA Heart Laser Catheter is introduced via the distal free end of the graft, into the connector, and is perpendicularly fixated by the ELANA Heart Fixation Device. The arteriotomy is lasered per excimer laser (**b**). The lower fork is opened and inserted into the lumen of the coronary artery, while the upper fork and ELANA Heart Fixation Device maintain compression on the graft ensuring proper positioning of the graft and ELANA Heart Laser Catheter (**c**). The ELANA Heart Clip is closed and the arteriotomy in the coronary wall is lasered. The ELANA Heart Fixation Device is removed and the ELANA Heart Laser Catheter is retracted, retracting the lasered piece of coronary artery wall per vacuum (**d**). A hemoclip is placed at the distal end of the graft. The ELANA Heart Clip serves as an implant (**e**)

The ELANA Heart Bypass creates a 0-degree side-to-side (functional end-to-side) anastomosis (Fig. 1). Large arteriotomies are commonly described for this type of anastomosis, which is most often used for a left internal mammary artery (LIMA) to left anterior descending coronary artery (LAD). Glineur et al. advise a coronary arteriotomy length of 3 times the diameter of the coronary artery and a graft opening size of 1.5 times the size of the coronary arteriotomy length [8]. For the ELANA Heart Bypass, with a standard arteriotomy size, no relation to the receiving coronary artery size and coronary hemodynamics was described so far. Except for two important requirements: (1) the arteriotomy width should not exceed the diameter of the coronary artery since the device should be introduced into the coronary artery; (2) the arteriotomy length should not exceed the diameter of the graft to fit the laser catheter for arteriotomy creation. To elaborate, whereas minimal arteriotomy sizes are recommended for the hand-sewn, the ELANA Heart Bypass requests a maximum arteriotomy size risking a relative stenosis.

In clinical practice, the hemodynamic significance of coronary stenoses is assessed by functional tests as fractional flow reserve (FFR). Under maximal hyperemic flow, the pressure ratio over the coronary stenosis is measured with the use of an intracoronary pressure wire [9]. Nowadays, FFR can be estimated using computational fluid dynamics (CFD) [10, 11]. CFD predicts fluid flow under predefined conditions, using physical balance of mass and momentum laws on a prescribed geometry [10, 12]. Since only limited resources are required, CFD is particularly useful in the first design phases of new (medical) devices.

We hypothesize optimal coronary hemodynamics to be achieved for an arteriotomy size as large as the cross-sectional area of the receiving coronary artery. Yet, we expect secondary influencers to eventually increase the resistance and thus to lower the FFR. In this study, we aim to gain insight into the coronary hemodynamics following ELANA Heart Bypass. We will therefore investigate the effect of varying the arteriotomy size and graft diameter on the CFD-derived FFR.

## Methods

A finite volume-based CFD model was used to mimic the hemodynamics directly after CABG with the ELANA Heart Bypass. The geometry of the entire graft and a small part of the coronary artery, the area near the bypass, was used as domain to compute the 3D fluid flow. The rest of the cardiovascular system was described by applying corresponding boundary conditions. Different simulations were performed with above mentioned predefined geometry and boundary conditions, varying the independent parameters; graft diameter (1.5–5.0 mm) and arteriotomy size (0.94–3.6 mm^2^). Varying these independent parameters will provide us insight into the influence of these parameters on the parameters of interest; distal coronary pressure (in mmHg), flow (in mL/min), and the CFD-derived FFR. The latter is calculated from inlet (aortic) and outlet (distal coronary) pressures at hyperemic flow. All simulations are performed for steady in-flow conditions. This is motivated by the observation that CFD-derived FFR yields the same value with steady and unsteady simulations because FFR is a metric that is based on time-averaged pressure values [13]. The computational, or numerical, model captured the qualitative trend of a set of experiments using a mock loop that applied flow through the ELANA Heart Bypass anastomosis. In modeling the coronary artery only (pre-surgery situation), no significant difference was found between numerical and experimental models. When modeling the ELANA Heart Bypass (post-surgery situation), absolute values differed between numerical and experimental models for both flow (resp. 238 IQR 37 vs. 170 IQR 50; *P* < 0.001) and pressure drop (resp. 54 IQR 7 vs. 48 IQR 9; *P* = 0.006). Since trends were consistent between the numerical and experimental models, the deviation in absolute values was accepted. Data is available in the supplementary materials (Online Resource 1).

### Endpoints

The primary endpoint defines optimal arteriotomy size. Distal coronary pressure (mmHg), flow (mL/min), and CFD-derived FFR are therefore plotted for a range of arteriotomy sizes. The secondary endpoint defines the influence of graft diameter. Distal coronary pressure (mmHg), flow (mL/min), and CFD-derived FFR are therefore plotted for a range of graft diameters.

### Geometry and Mesh

**Fig. 2:**
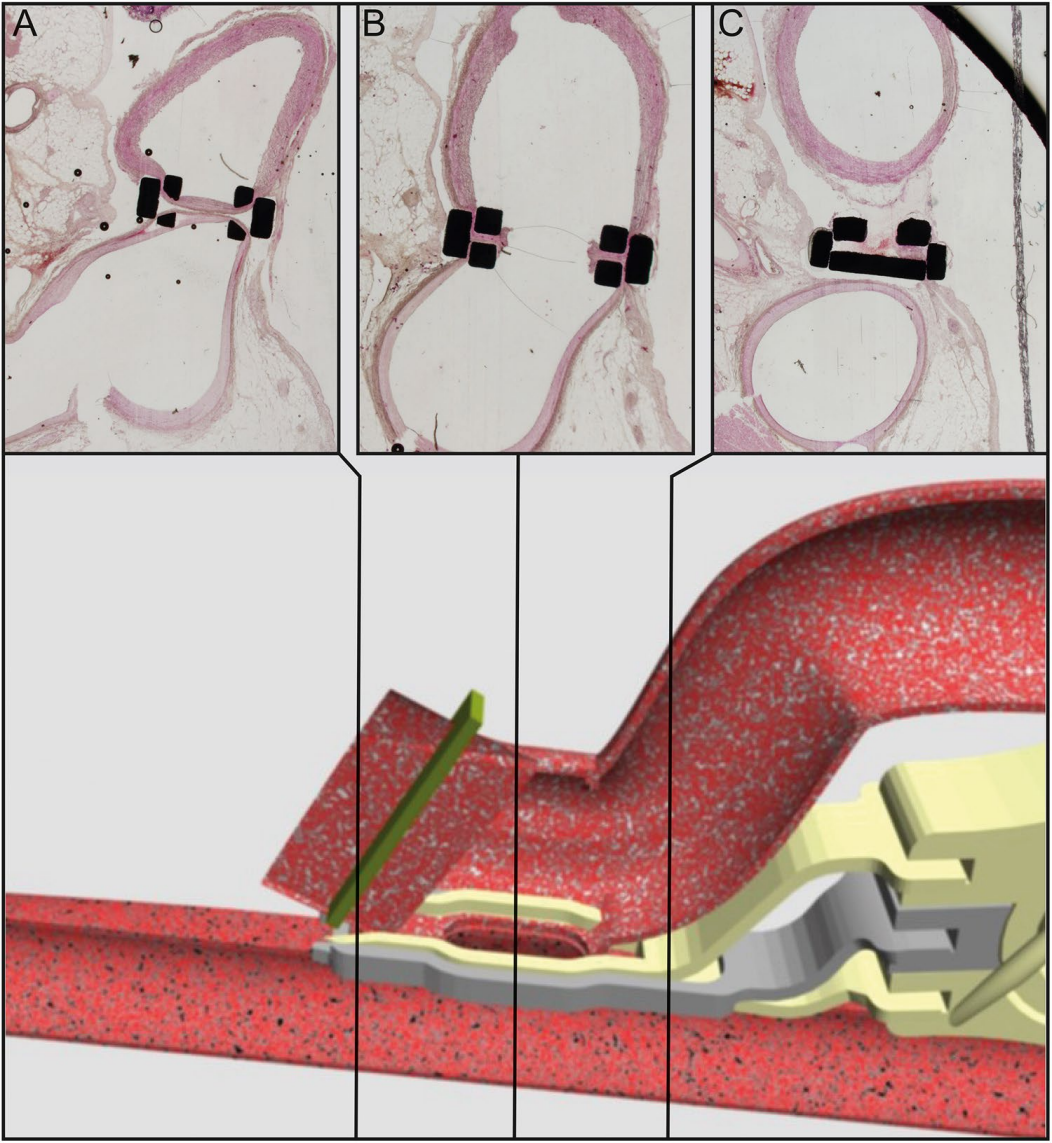
ELANA Heart Bypass anastomosis. The titanium ELANA Heart Clip connects graft and coronary artery by the intraluminal positioned pins; 0-degree, side-to-side, in adventitia-adventitia apposition. To indicate part of the geometry sources, we depict three transverse H&E stained sections of the situation directly postoperative in a porcine LIMA-LAD ELANA anastomosis; LIMA is depicted above, LAD below [7]. Middle black squares are the titanium pins of upper and lower forks (yellow in the simulation below), and lateral black rectangles are the ring for lateral compression (grey in the simulation below). The distal end of the graft is occluded by a hemostatic clip and thus forms a functional end-to-side anastomosis. The ELANA anastomosis is oval-shaped. Anastomotic toe/outflow (**a**), mid-anastomosis (**b**), anastomotic heel/inflow (**c**)

**Fig. 3:**
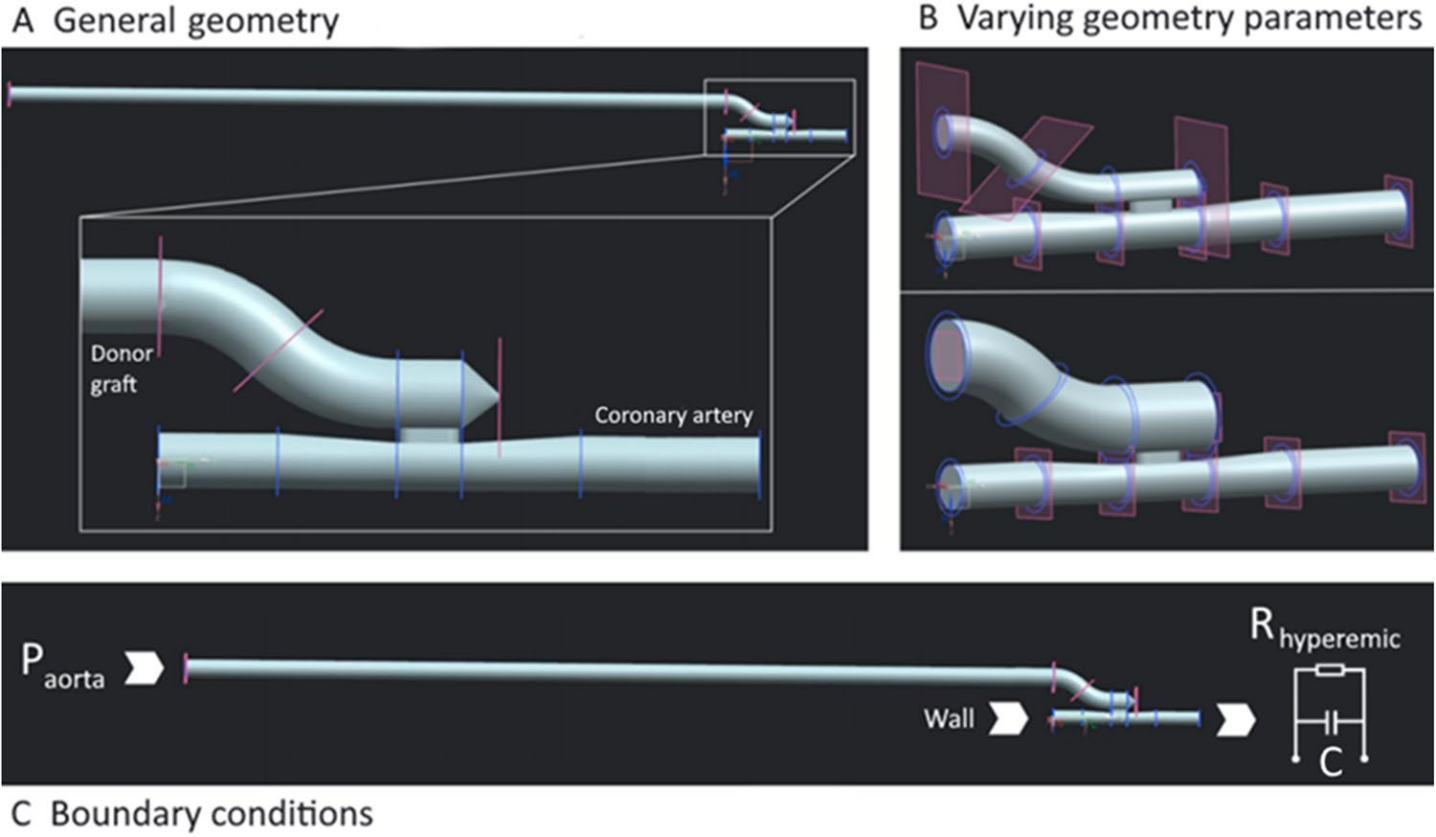
Schematic overview of the most important features of the numerical model. 3D fluid geometry of the ELANA Heart Bypass anastomosis, including the graft (**a**). The effect of changing one of the geometry parameters (increase in graft diameter) on the geometry (**b**). Overview of the various boundary conditions of the model (**c**)

The geometry for this study was designed with SIEMENS NX 8.0 software (Siemens, Munich, Germany). The geometry was reconstructed from histological sections of previous acute porcine experiments to the ELANA Heart Bypass, as is depicted in Fig. 2 [7]. The designed geometry is displayed in Fig. 3a. The graft length is estimated at 12 cm, as was measured from coronary angiography during consecutive porcine trials to the ELANA anastomosis (approved by the animal ethics committee, AVD1150020185105-01). To create perspective, the maximum available human LIMA-graft length is 20.7 ± 2.1 cm [14, 15]. The inner diameter of the distal coronary artery is set at 1.85 mm, similar to the mean distal human LAD [16].

The independent parameters, graft diameter and the arteriotomy size, were varied in each simulation as is presented in Table 1.

**Table 1:** Variable geometry parameters

Simulation	Graft diameter, mm	Arteriotomy size, mm^2^
1	**1.5**	1.434
2	**2**	1.434
3	**2.5**	1.434
4	**3**	1.434
5	**4**	1.434
6	**5**	1.434
7	2.5	**0.943**
8	2.5	**2.085**
9	2.5	**2.811**
10	2.5	**3.342**
11	2.5	**3.639**

Graft diameter was varied in simulations 1–6. Arteriotomy size was varied for simulations 7–11. All simulations included a 12 cm graft length, 1.85 mm diameter coronary artery (2.69 mm^2^ cross-sectional area), 97 mmHg inlet pressure, 3.8 × 109 P·s·m^−3^ microvascular resistance

Note in Fig. 2 that the arteriotomy has an oval shape. The arteriotomy size is limited by the excimer laser catheter shape that needs to fit the distal end of the graft, thus in all simulations, the arteriotomy length is chosen smaller than the graft diameter. The anastomotic width is limited by the intraluminal positioning of the titanium pins. By varying the length of this oval-shaped anastomosis, large arteriotomy sizes could be achieved even in combination with small receiving coronary arteries. The effect of the arteriotomy size on the coronary hemodynamics was studied by varying this length (resulting in arteriotomy sizes between 0.94 and 3.64 mm^2^), while the graft diameter was kept constant (2.5 mm) [17].

To investigate the effect of the graft size on coronary hemodynamics, the graft diameter was varied (different values between 1.5 and 5 mm were chosen), while the arteriotomy size (1.43 mm^2^) conformed with the current ELANA Heart Bypass.

Figure 3 b demonstrates how varying one of these parameters, the graft diameter, in this case, influences the geometry in the simulation.

Once the geometry was designed, the internal volume was meshed into small elements for CFD analyses. An inflation layer was added to the mesh to accurately capture the fluid dynamics of the boundary layer region of the vessel. In addition, the element size near the anastomosis was refined, to create more detail in this crucial part of the mesh. It is important to mention that a mesh sensitivity study was performed on the smallest developed geometry. From this mesh sensitivity study, an optimal mesh element size was chosen (global element size: 2 × 10^–4^ m, element size near anastomosis: 1.4 × 10^–4^ m). The same element size was also applied to the larger geometries. Even though this leads to more computational complex meshes than needed, it saved a lot of time due to faster mesh development.

### Inlet Boundaries

Following CABG, two coronary inlets exist: the native coronary artery inlet and the graft inlet. However, we decided to model the worst-case scenario considering coronary perfusion, namely full occlusion of the native coronary artery in which all flow enters the coronary artery via the anastomosis and thus might cause the highest local pressure drop. The coronary artery inlet is therefore modeled as a rigid wall (Fig. 3c). No-slip boundary conditions (i.e., no velocity of fluid relative to the boundary) were applied to the wall.

At the graft inlet, on the contrary, a pressure of 97 mmHg was prescribed (Fig. 3c). Values for the pressure inlet were chosen to represent physiologic human aortic pressure [13, 18].

### Microvascular System: Windkessel Model

In our model one outlet exists, namely the distal coronary artery. In vivo, this artery merges into the microvascular system. A two-element Windkessel model is prescribed to the distal outlet of the coronary artery of our model to describe this microvascular system (Fig. 3c). The Windkessel model is based on the analogy between electrical and fluid parameters and it considers the vascular system as a parallel combination of arterial compliance *C* and peripheral resistance *R*. Due to the constant pressure prescribed to the graft inlet of our model, the simulations have a constant flow. Thus, using more complex coronary Windkessel models such as the model of van der Horst instead of a simple two-element Windkessel model is not necessary [19].

Two parameters need to be determined for the two-element Windkessel model: microvascular resistance and compliance. The total capacitance of the microvascular system is set at a value of 2.13 × 10^−9^ *m*^3^ Pa^−1^ [20]. Usually, the resistance of the microvascular system (1.3 × 10^10^ P s m^−3^) is calculated using Ohms law (Eq. 1), the mean distal coronary flow (59 ± 15.0 mL/min) and the aortic pressure (97 mmHg) [21]:

1 $$R=\frac{p}{q}$$

However, standard clinical FFR measurements require a hyperemic state, which is induced by nitroglycerin and adenosine injection [22]. This injection thus diminishes microvascular resistance by dilation of the microvascular vessels and thereby initiates a direct increase in flow with a factor of 3.3–4 [13, 23, 24]. This factor is called the hyperemic factor (*f*_hyp_). In order to calculate the FFR, the hyperemic flow was modeled by applying a hyperemic factor of 3.5. Using Ohms law (Eq. 1), but now with the hyperemic flow (3.5 × 59 = 207 mL/min) and the aortic pressure (97 mmHg), results in a microvascular resistance of 3.8 × 10^9^ P s m^−3^.

### Fluid Model

The blood flow in the computational model is described by the Navier–Stokes equations [25, 26]. These equations are solved by using the commercial software ANSYS Fluent 2020 R1 (ANSYS, Inc., Cannonsburg, MI, USA) [27]. Gravity is not included in any of the calculations. In the coronary arteries, typical shear rates have values above 100 s^−1^. For these high shear rates, the blood viscosity is almost constant [28]. Therefore, blood is modeled as a Newtonian (*μ* = constant = 0.035 Poise) and continuous fluid, as well as incompressible (*ρ* = constant = 1050 kg/m^3^). Moreover, the vessel wall was modeled as a rigid wall, where no-slip boundary conditions were applied.

Given the abovementioned blood viscosity and density, the Reynolds number (as defined in Eq. 2) in the coronary arteries is below 1000, even when considering a high velocity (*V*) of 1.5 m/s with a 1.85 mm vessel diameter (*D*_h_). Therefore, the flow has been assumed to be laminar.

2 $$\mathrm{Re}=\frac{\rho V{D}_{\mathrm{h}}}{\mu }$$

## Results

**Fig. 4:**
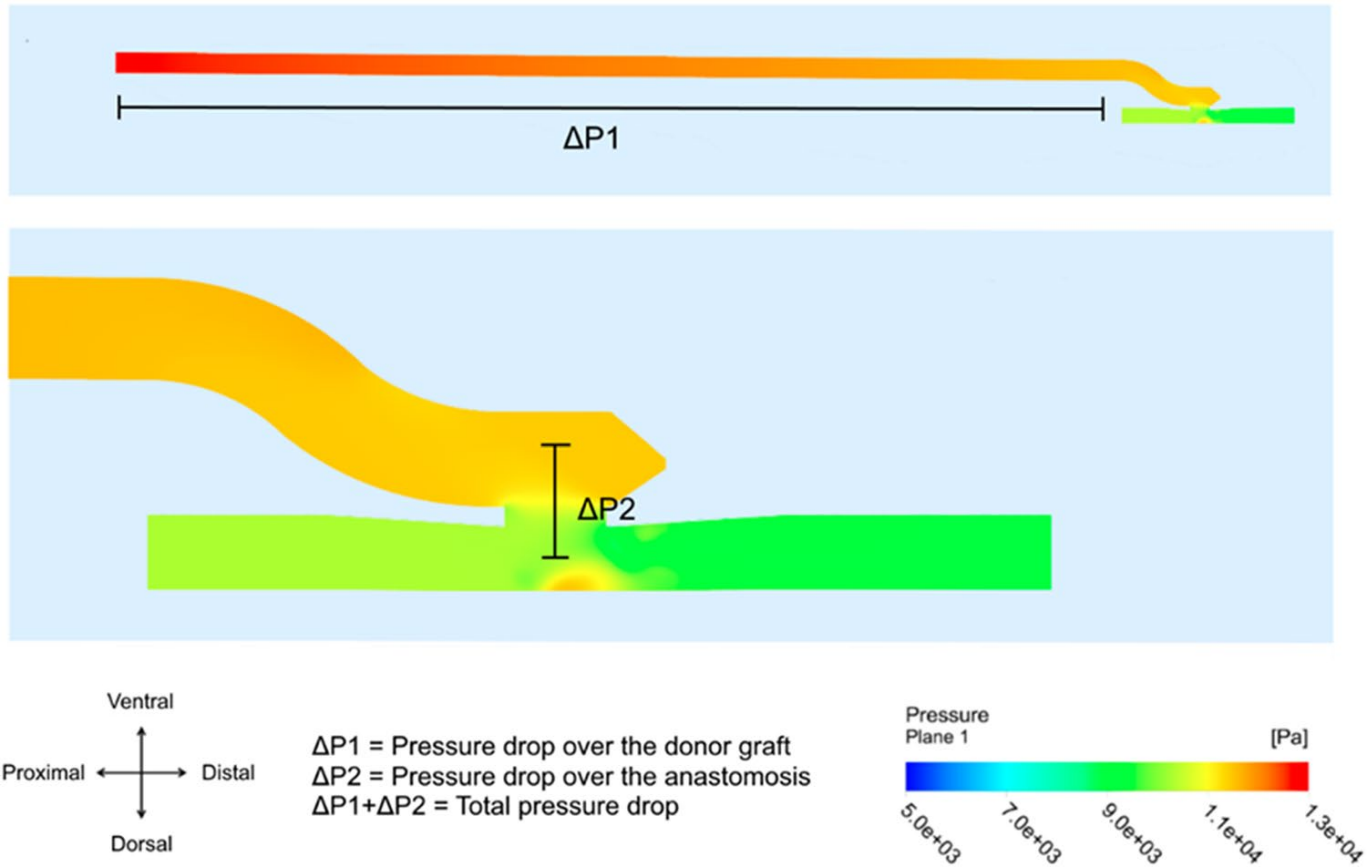
Pressure in graft and coronary artery for the ELANA anastomosis. The two defined pressure drops are depicted. The bottom image presents the enlargement, with a focus on the anastomosis, of the total simulation presented above. Arteriotomy size = 1.43 mm^2^, graft diameter = 2.5 mm, coronary diameter = 1.85 mm

Figure 4 presents a pressure plot generated by the finite volume model. The plot shows the two most important effects of the bypass on coronary hemodynamics, as detected by the model, namely (1) a pressure drop in the anastomotic construction itself and (2) a gradual pressure drop over the entire length of the graft. We describe the two pressure drops together as the total pressure drop.

### Arteriotomy Size

**Fig. 5:**
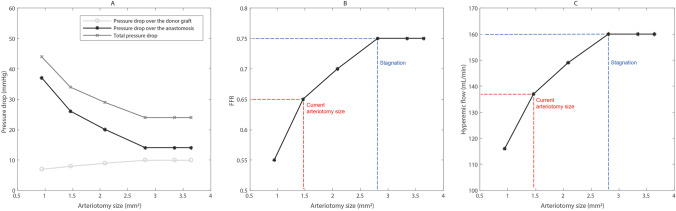
Results of the pressure drop (**a**), FFR (**b**), and hyperemic flow (**c**) for a range of arteriotomy sizes. Graft diameter = 2.5 mm, coronary diameter = 1.85 mm

Figure 5 presents the plots of pressure (Fig. 5a), CFD-derived FFR (Fig. 5b), and flow (Fig. 5c) for the simulated arteriotomy sizes. Note that these simulated values are based on a coronary diameter of 1.85 mm, a graft diameter of 2.5 mm, an inlet pressure of 97 mmHg, and the predefined microvascular resistance.

Figure 5 b shows that the current size ELANA Heart Bypass, with an arteriotomy size of 1.43 mm^2^, resets in an estimated FFR value of 0.65. According to Fig. 5c, the corresponding hyperemic flow for this arteriotomy size is 137 mL/min.

Figure 5 a shows that a larger arteriotomy is related to an increase in graft-related pressure drop and a decrease in the localized anastomotic pressure drop. The combination of these effects led to a decrease in total pressure drop and an increase in FFR (Fig. 5b), regarding a larger arteriotomy. Figure 5 c depicts increasing flow in simulations with larger arteriotomy sizes.

It must be noticed that the steepness of all curves in Fig. 5 decreases with larger arteriotomies. The graphs in Fig. 5 all stagnate once an arteriotomy size of 2.80 mm^2^ is reached. In other words, the outcome parameters (pressure, FFR, flow) stagnate when the arteriotomy size surpasses the cross-sectional area of the coronary artery (2.69 mm^2^). The FFR stagnates at a value of 0.75 (Fig. 5b) and the hyperemic flow at 160 mL/min (Fig. 5c).

### Graft Diameter

**Fig. 6:**
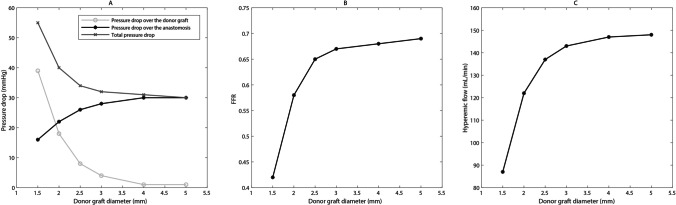
Results of the pressure drop (**a**), FFR (**b**), and hyperemic flow (**c**) for a range of graft diameters. Arteriotomy = 1.43 mm^2^, coronary diameter = 1.85 mm

Figure 6 presents the plots of pressure (Fig. 6a), CFD-derived FFR (Fig. 6b), and flow (Fig. 6c) for the simulated graft diameters. Note that these simulated values are based on a coronary diameter of 1.85 mm, the current ELANA Heart Bypass with an arteriotomy size of 1.43 mm^2^, an inlet pressure of 97 mmHg, and the predefined microvascular resistance.

Figure 6 a shows that larger graft diameters are related to a decrease in pressure drop over the graft itself. Yet, it is also related to an increase in pressure drop localized over the anastomosis. An increase in the graft diameter is associated with a decrease in the total pressure drop and an increase in FFR (Fig. 6b). In addition, Fig. 6c indicates that the coronary flow increases with increasing graft diameters.

As depicted in Fig. 6, the steepness of all curves decreases with the increase in graft diameter. Ultimately, with a graft diameter of 5 mm, the FFR approximates 0.70 (Fig. 6b) and the hyperemic flow 150 mL/min (Fig. 6c).

## Discussion

To gain insight into the coronary hemodynamics following CABG using ELANA Heart Bypass, we estimated CFD-derived FFR within this study. The results of this study clearly depict that application of the current ELANA Heart Bypass (arteriotomy size 1.43 mm^2^) on a coronary artery of 1.85 mm diameter (2.69 mm^2^ cross-sectional area) causes a pressure drop. The pressure drop within the anastomosis is minimized in case the arteriotomy size surpasses the cross-sectional area of the coronary target. Still, FFR at this point is 0.75. This suggests the influence of other resistances within the bypass route, gradually over the full graft length as was identified, and, possibly, due to the curvature of the anastomosis.

To elaborate, the current oval shape of the ELANA Heart Bypass creates a limited arteriotomy size in relation to the required minimal coronary artery size*.* The ELANA requests a minimum coronary artery diameter in order to insert the pins of the ELANA Heart Clip (Figs. 1 and 2). The minimum coronary diameter requested for the current design is 1.4 mm (i.e., cross-sectional area 1.54 mm^2^), indicating that the arteriotomy (1.43 mm^2^) is smaller than the cross-sectional area of the receiving coronary artery. The question remains whether the resulting pressure drop is clinically relevant.

### Fractional Flow Reserve

Since the current role of FFR is quantification of stenosis severity in the native vessel for optimal planning of revascularization strategies, literature of FFR following CABG is limited. It is well-defined that FFR-guided revascularization with a cut-off value of ≤ 0.80 improves clinical outcomes for both PCI and to a lesser extent CABG [29–32]. Of note, FFR measurements are also used to assess the degree of coronary and bypass graft stenoses, and therefore decision-making regarding PCI in bypass graft stenoses [33]. Abundant, and most significant, clinical evidence for FFR-guided revascularization is thus based on patients undergoing PCI. Notwithstanding the lack of direct clinical evidence, one might expect a functional coronary bypass anastomosis to result in improved FFR values with a FFR > 0.80 as safe cut-off value. Subsequently, physical conservation laws themselves do not distinguish stenoses and anastomoses. CFD-derived FFR is insightful since it illustrates the effect of flow impairments per variation in local geometry. In translation to the complete picture, one aims to guarantee myocardial blood flow in line with the distal myocardial demand in rest and exercise, which is determined by volume.

During the design phases and preclinical evaluations of various prototype anastomotic connectors, FFR has been measured. In a porcine off-pump CABG LIMA-LAD with complete proximal LAD-ligation, Suyker et al. presented a FFR 0.93 for the S2 connector and 0.96 for the hand-sewn anastomoses 90 days postoperative [34]. Stecher et al. presented a FFR 0.82 directly postoperative for a larger ELANA prototype (2.6-mm^2^ arteriotomy and 4.5-mm^2^ cross-sectional area of the coronary target) [5]. Pilot experiments in the same setup with the ELANA Heart Bypass were complex because of flow disturbances and difficult catheter routing. Final validated FFR values measured were 0.77 and 0.68 [non-published data].

Notwithstanding the FFR, graft flow per perioperative transit time flow measurement (TTFM) is measured as a standard of care post anastomotic construction [35]. The associated hyperemic flow (160 mL/min at maximum FFR 0.75) presented in our model is supposedly more than sufficient following CABG [36]. The discrepancy between FFR and flow implicates that the narrowing caused by the ELANA anastomosis is not clinically relevant. And while this is the case, there are two important notes in the interpretation of these values: (1) flow depicts the total system but not specifically local geometry; (2) the absolute values of the model are influenced by its input variables.

### Model Parameters

With respect to computational models, it is essential to understand that the absolute output values should be reflected carefully. This particular model is mainly constructed with literature-based input variables, resulting in considerable variances. Microvascular resistance, for instance, depends on the prescribed flow (59.1 ± 15.0 mL/min) and hyperemic factor (flow multiplication by 3.3–4) [13, 23, 24]. Given an aortic pressure of 97 mmHg and Ohms law, these variations may lead to a microvascular resistance varying from ${2.6 \times 10}^{9}$ to ${5.3 \times 10}^{9}$ P·s·m^−3^. In the current model, a resistance of 3.8 × 10^9^ P·s·m^−3^ was applied. However, the application of a different hyperemic factor or flow has a large impact on microvascular resistance, with a direct reflection in FFR. Moreover, the LAD flow from literature (59.1 ± 15.0 mL/min) might overestimate the actual flow found in the distal area of the LAD, since in the distal part of the vessel, the flow is divided between the LAD and its side branches. Other factors do variate between or within patients, for example, the inlet pressure. The inlet pressure of the graft prescribed in this model was 97 mmHg. Since the current numerical model describes the coronary fluid dynamics during hyperemia, and thus maximum available flow with consistent microvascular and anastomotic resistances, we may assume that the FFR is not affected by this change in inlet pressure [37].

Most important, however, was the validation of the computational model to an experimental setup. This validation was complicated by the fact that only a prototype device was available at this time. Whereas the ELANA Heart Bypass creates two exactly opposing arteriotomies, the prototype used was not yet able to create complete arteriotomy at the receiving coronary artery site. We therefore manipulated the anastomosis to manually remove the remaining vessel wall tissue. Opening the anastomosis could have shifted the arteriotomies, resulting in decreased anastomotic size. Tearing the vessel wall tissue could have enlarged or decreased the anastomosis size by either removing too much or too little. Significant differences were detected between the experimental and computational models. These differences were accepted since the general trends remained consistent. Thus, the current numerical model is successful in describing the general hemodynamic trends and in depicting the local flow impairments by the anastomotic geometry. The absolute values of the output parameters, however, should be taken with caution.

Then, there are variables that will differ per patient and per created bypass, for example, the amount of competitive flow via the native coronary artery, graft length, and graft diameter. The volume of competitive flow is essential within the calculation. Any perfusion via the native coronary system will add to the total perfusion in the distal coronary artery and will therefore increase the FFR. The situation currently modeled had no native coronary perfusion and could be stated the worst case, regarding FFR. We did model this worst-case situation since the anastomosis should also function in (sub)total coronary occlusions. In addition, it is well-known that the patency of the native system is limited over time due to competitive graft flow. On the other hand, the graft and anastomotic patency would be compromised if competitive flow via the native coronary system takes the upper hand [38]. Regarding the graft, the indicated gradual pressure drop is more extensive in grafts with smaller diameters and, presupposed, longer grafts. It is well known that grafts tend to remodel following CABG, often leading towards increased diameters, especially for the in-situ arterial grafts. Based on our findings we expect graft remodeling to positively affect the FFR in the longer term. Thus, currently presented FFR values of the direct post-operative situation might deviate from the FFR in the longer term. More patient-specific computational models can aid in the prediction of the impact of this remodeling on the FFR and the other patient-specific conditions.

### Future Perspectives

Notwithstanding the broad range of variables described above, trends are well-recognized within this study and indicate that application of a small arteriotomy might induce a risk in CABG. Whereas clinical relevance is not yet defined, we recommend future studies and reluctance for the use of the ELANA Heart Bypass on areas with a large run-off until then. Future studies could focus on the match between the device size and the coronary target area, where it was already intended to create a variety of devices with the current device being the smallest.

Eventually, the arteriotomy size of the ELANA Heart Bypass could be increased in proportion to the coronary target diameter and by upgrading the device in its length. Future studies could increase model reliability by optimization of input variables and patient-specific applications. Coronary target sizes could be varied, as could coronary inlet pressures to model the effect of subtotal coronary stenoses or competitive flow [39]. Ultimately, patient-specific models could predict the minimally requested arteriotomy size for the ELANA Heart Bypass.

## Conclusion

The constructed CFD-derived FFR indicates both the arteriotomy and the graft diameter in the anastomotic construction to influence the coronary hemodynamics. Increasing the graft diameter and/or arteriotomy size results in improved coronary hemodynamics. A maximum improvement is reached in arteriotomy size once the area of arteriotomy surpasses the cross-sectional area of the coronary artery. The ratio between the required minimal coronary diameter for application and the ELANA arteriotomy size effectuates a pressure drop that could be clinically relevant. Additional research and eventual lengthening of the anastomosis is advised.

## Supplementary Information

Below is the link to the electronic supplementary material.

Supplementary file1 (PNG 164 KB)

Supplementary file2 (PNG 412 KB)

Supplementary file3 (PNG 154 KB)

Supplementary file4 (PNG 165 KB)

Supplementary file5 (PDF 504 KB)

## Data Availability

Data is available in the supplementary file. Extra details could be requested via the author Hanneke Crielaard (h.crielaard@erasmusmc.nl), as is stated in the supplementary file.

## Abbreviations

CABG: Coronary artery bypass grafting
CFD: Computational fluid dynamics
FFR: Fractional flow reserve
LAD: Left anterior descending coronary artery
LIMA: Left internal mammary artery
PCI: Percutaneous coronary intervention

## Declarations

### Animal Studies Statement

Information was obtained out of previous and current animal studies [7]. All institutional and national guidelines for the care and use of laboratory animals were followed and approved by the appropriate institutional committees (2013.II.08.094) (AVD1150020185105-01).
