# Supplementary figures and images for: Evaluating the Arteriotomy Size of a New Sutureless Coronary Anastomosis Using a Finite Volume Approach
Source: J Cardiovasc Transl Res. 2023 Mar 21;16(4):916–26. doi: 10.1007/s12265-023-10367-9 (PMC10480236; doi:10.1007/s12265-023-10367-9)

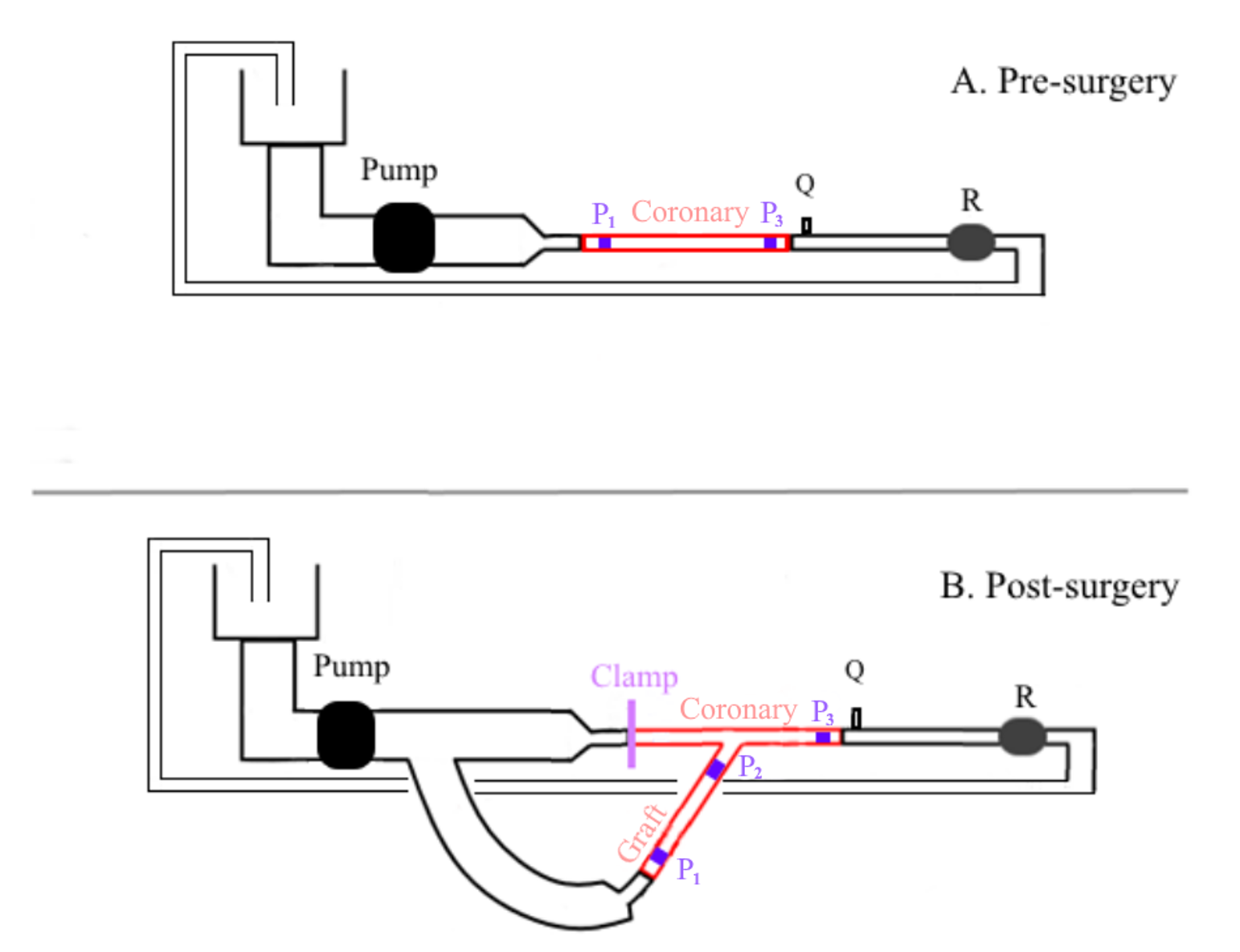

Supplement: Supplementary file 1 — Supplementary file1 (PNG 164 KB) [file 12265_2023_10367_MOESM1_ESM.png]

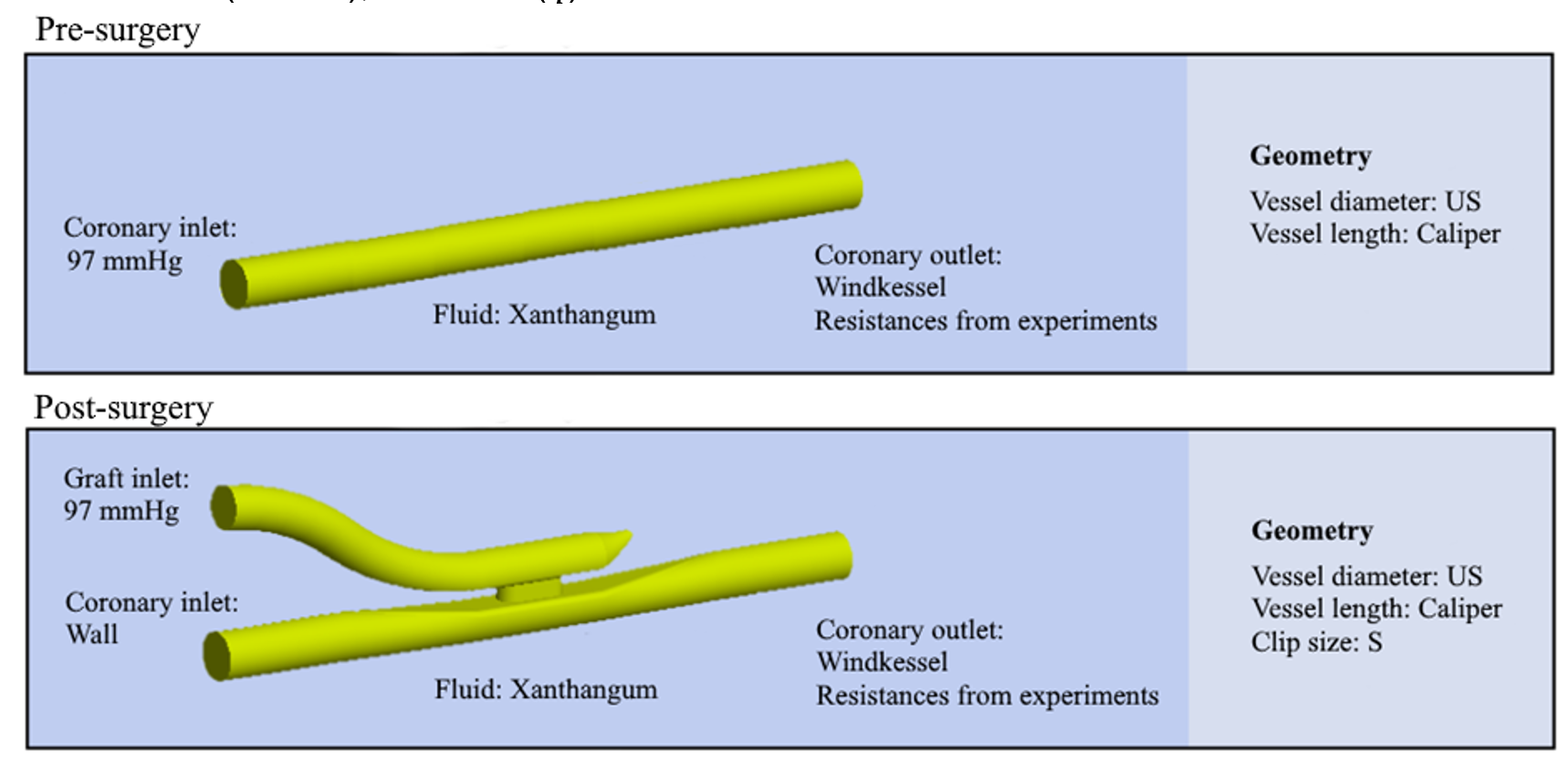

Supplement: Supplementary file 2 — Supplementary file2 (PNG 412 KB) [file 12265_2023_10367_MOESM2_ESM.png]

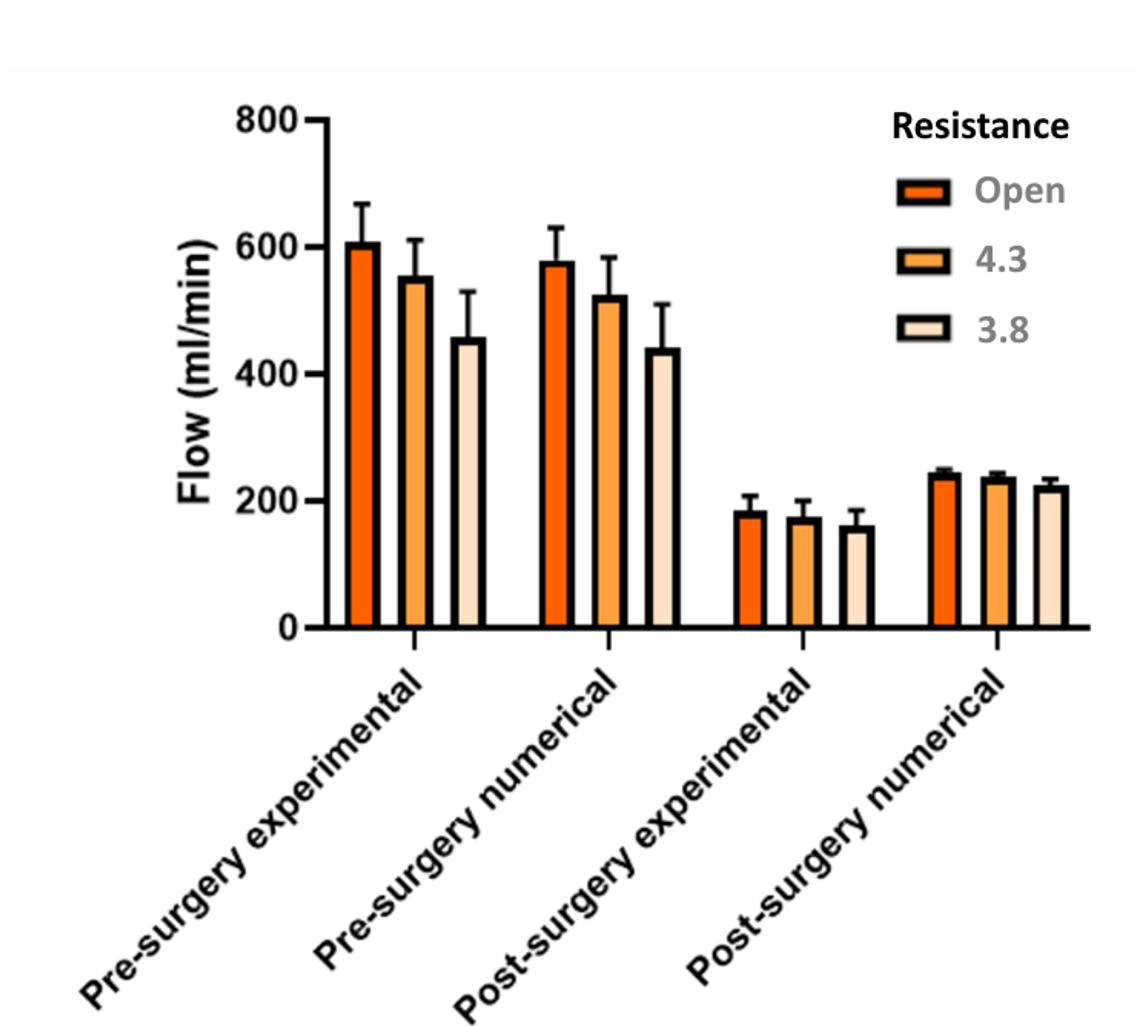

Supplement: Supplementary file 3 — Supplementary file3 (PNG 154 KB) [file 12265_2023_10367_MOESM3_ESM.png]

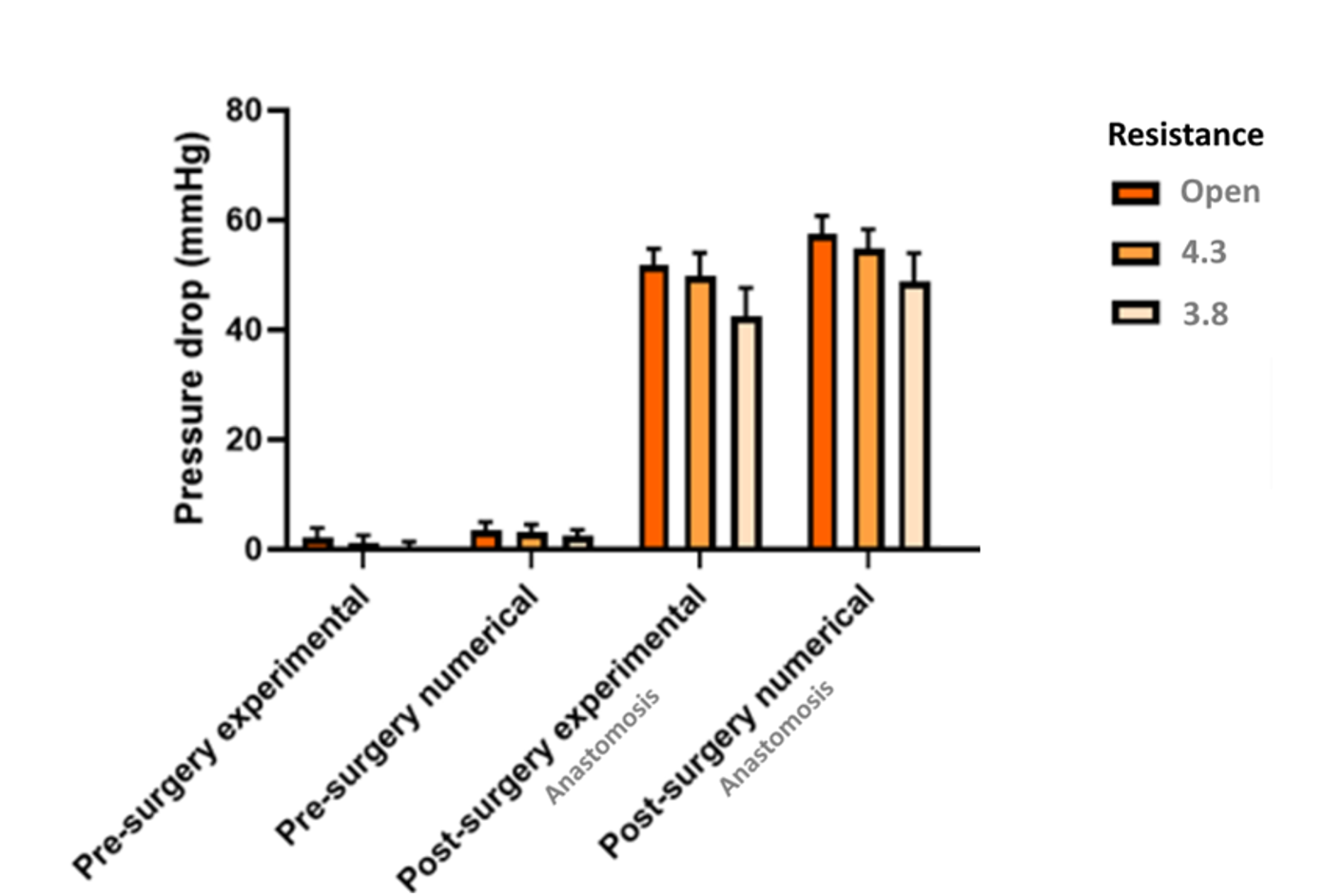

Supplement: Supplementary file 4 — Supplementary file4 (PNG 165 KB) [file 12265_2023_10367_MOESM4_ESM.png]
