# Supplementary material for: Evaluating the Arteriotomy Size of a New Sutureless Coronary Anastomosis Using a Finite Volume Approach
Source: J Cardiovasc Transl Res. 2023 Mar 21;16(4):916–26. doi: 10.1007/s12265-023-10367-9 (PMC10480236; doi:10.1007/s12265-023-10367-9)
Supplement: Supplementary file 5 — Supplementary file5 (PDF 504 KB) [file 12265_2023_10367_MOESM5_ESM.pdf]

Supplementary information to:

EVALUATING THE ARTERIOTOMY SIZE OF A NEW SUTURELESS CORONARY ANASTOMOSIS USING A FINITE ELEMENT APPROACH

Journal of Cardiovascular Translational Research

Hanneke Crielaard MSc[1,2,3*], Marieke Hoogewerf MD[4,5*], Bart P. van Putte MD PhD[5,6], Frans N. van de Vosse PhD[2], Georgios J. Vlachojannis MD PhD[4], David Stecher MD PhD[7], Marco Stijnen PhD[1], Pieter A. Doevendans MD PhD[4,8].
* The authors Hanneke Crielaard and Marieke Hoogewerf contributed equally to the work.

1. LifeTec Group, Eindhoven, The Netherlands
2. Department of Cardiovascular Biomechanics, University of Eindhoven, Eindhoven, The Netherlands
3. Department of Biomedical Engineering, Erasmus Medical Centre, Rotterdam, The Netherlands
4. Department of Cardiology, University Medical Center Utrecht, Utrecht, The Netherlands
5. Department of Cardiothoracic Surgery, St. Antonius Hospital, Nieuwegein, The Netherlands
6. Department of Cardiothoracic Surgery, Amsterdam University Medical Center, Amsterdam, The Netherlands
7. Department of Cardiothoracic Surgery, University Medical Center Utrecht, Utrecht, The Netherlands
8. Netherlands Heart Institute, Utrecht, The Netherlands

Corresponding author:
Hanneke Crielaard
Department of Biomedical Engineering, Erasmus Medical Center
Doctor Molewaterplein 40, 3015 GD, The Netherlands
Tel: +31(0)6 3643 4552
Mail: h.crielaard@erasmusmc.nl

METHODS

Six mock loop experiments were performed to validate the numerical model. For each mock-loop experiment, both the pre-surgery (before ELANA Heart Bypass) and the post-surgery (after ELANA Heart Bypass) situation were mimicked. For each mock loop experiment, numerical simulations of these experiments were executed with the same input parameters as applied in the experiments. The output parameters of the experimental and the numerical model were compared.

Biological material

Porcine urethrae (Dutch female Landrace hybrid pigs +/- 110 KG) were used to mimic the coronary vessel and the donor graft in the mock loop experiments. The wall thickness of the urethrae is approximately similar to the wall thickness of the porcine left internal mammary artery (LIMA) and therefore suits application of the ELANA Heart Clip. Urethrae were chosen instead of the porcine LIMA for their bigger diameter, to fit the cannulas of the mock loop system, and for their lack of side branches. The urethrae were retrieved from the slaughterhouse and stored at -20°C until usage.

Mock loop set-up

A closed-loop pumping system was developed to mimic the hemodynamics before and after surgery with the ELANA Heart Bypass (Figure S1). First, one urethra was connected to the pumping system to mimic the coronary artery (pre-surgery situation, Figure S1.A). Afterwards, an additional urethra was attached to the system to mimic the donor graft. The ELANA Heart Bypass was executed conform the instructions for use drafted by AMT Medical BV. Full occlusion of the urethra representing the native coronary artery was mimicked by occluding the proximal side of this vessel with a hose clamp (post-surgery situation, Figure S1.B). In both the pre-surgery and the post-surgery situation, a bench vice was attached at the distal end of the tubing system, representing the resistance of the microvascular system. Three resistances were mimicked; fully open, 4.3 mm opening, and 3.8 mm opening. 10 Liter xanthangum (0.5 g/L) was infused into the set-up, mimicking the viscosity (3.71 mPa) of blood (hematocrit 34%). A stationary flow through the vessels was induced by a pump.

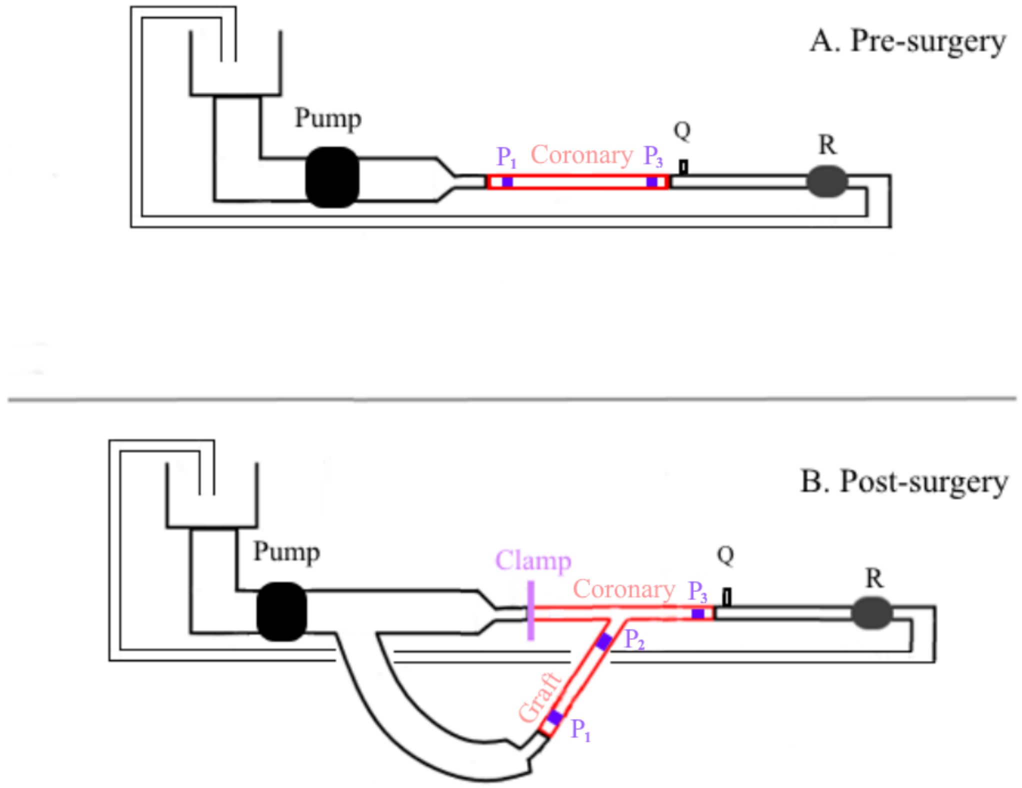

Fig. S1 Mock loop experiment to validate the numerical model. The pump can be controlled to make sure that the inlet pressure (P_1) is similar to the aortic pressure (97 mmHg). Pre-surgery (a): The pressure drop over the coronary artery (P_1 - P_3) and the flow (q) are measured. Post-surgery (b): The pressure drop over the graft (P_1 - P_2), the pressure drop over the anastomosis (P_2 - P_3), and the flow (q) are measured.

Measurements

Pressure wires (Certus, St Jude Medical, USA) were inserted into the urethrae to different locations (Figure S1). For the pre-surgery situation, these locations correspond to the proximal (P_1) and the distal (P_3) coronary artery. For the post-surgery situation, these locations correspond to the proximal (P_1) and the distal (P_2) graft, and the distal coronary artery (P_3). The driving pressure of the pump was adjusted to induce a physiological pressure value at the inlet of the coronary artery (pre-surgery) or the inlet of the graft (post-surgery). This pressure (P_1) was thus prescribed to be similar to the aortic pressure (97 mmHg).

The outlet pressures were measured (P_2 and P_3). From these outlet pressures, the pressure drop over the coronary artery was calculated for the pre-surgery situation. Also the pressure drop over the graft (P_1 - P_2) and the pressure drop over the anastomosis (P_2 - P_3) were calculated for the post-surgery situation.

Simultaneously to the pressure measurements, a flow sensor (2PXL, Transonic, USA) was used to measure the flow in the vessel corresponding to the distal coronary artery. All measurements were performed for all three resistances (completely open, 4.3 mm opening, and 3.8 mm opening). Finally, the geometric information of the vessels under hydrostatic pressure was gathered. The inner vessel diameter and the wall thickness were obtained using epicardial ultrasound (Medistim MiraQ ECUS, coronary setting). The length of the urethrae was measured using a caliper.

Numerical models

The geometry data obtained during the experiments was used to create six pairs of 3D fluid geometries. Each pair consisted of two geometries of which one mimicked the pre-surgery and the other the post-surgery situation of the experiments. The 3D geometries were used for numerical simulations in which also other parameters were chosen to represent the experiments (e.g. the viscosity of the xanthangum, the resistance applied by the bench vise) (Figure S2). The numerical model was used to calculate the outcome parameters measured in the mock loop. For the pre-surgery situation, these outcome parameters were the pressure drop over the coronary artery (P_1 - P_3) and the flow (q). For the post-surgery situation, these parameters included the pressure drop over the graft (P_1 - P_2), pressure drop over the anastomosis (P_2 - P_3), and flow (q).

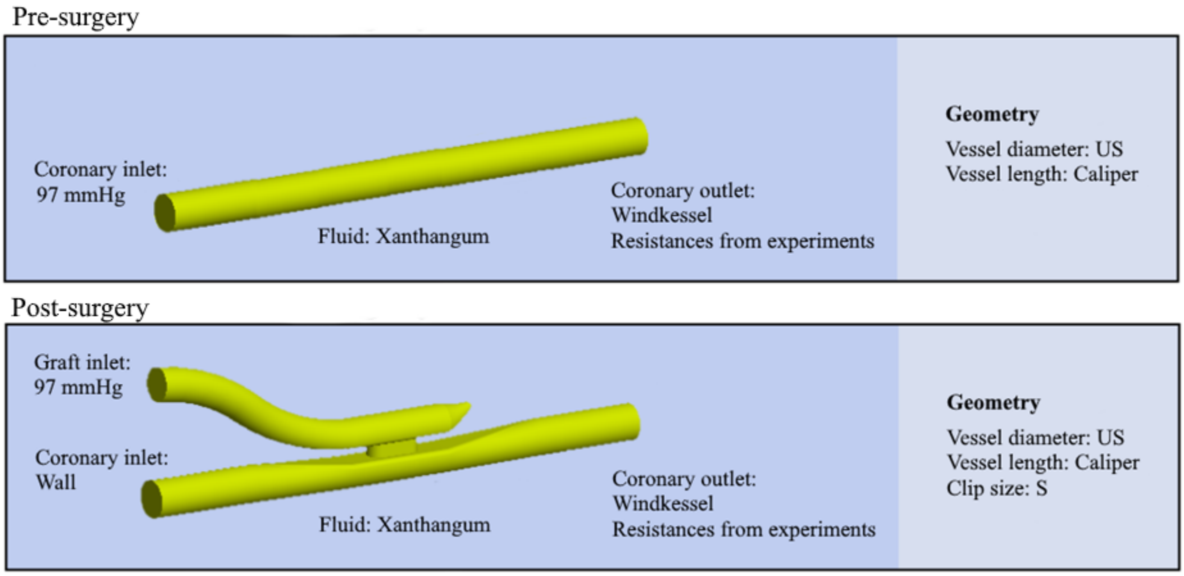

Fig. S2 Parameters used for validation of the numerical model.

RESULTS

We found that the general trends in the numerical and experimental data were similar, as is presented in the figures S3 for flow and S4 for pressure drop. Highest flow and pressure drop were measured at lowest resistance (i.e. open), as expected. Moreover, both the experiments and the numerical model showed that application of the ELANA Heart Bypass resulted in a decrease in flow and an increase in the pressure drop when comparing pre- and post-surgery situations. For the pre-surgery situation, no significant differences were found in between the numerical model and the experimental setting for both flow and pressure drop. For the post-surgery situation, both in the numerical model and in the experiments no pressure drop was found over the urethra representing the donor graft.

However, the absolute values of flow and the pressure drop over the anastomosis differed, with a higher median in the numerical model than in the experimental setting for both flow (resp. 238 IQR 37 vs. 170 IQR 50 ; $P < 0.001$) and pressure drop (resp. 54 IQR 7 vs. 48 IQR 9 ; $P = 0.006$).

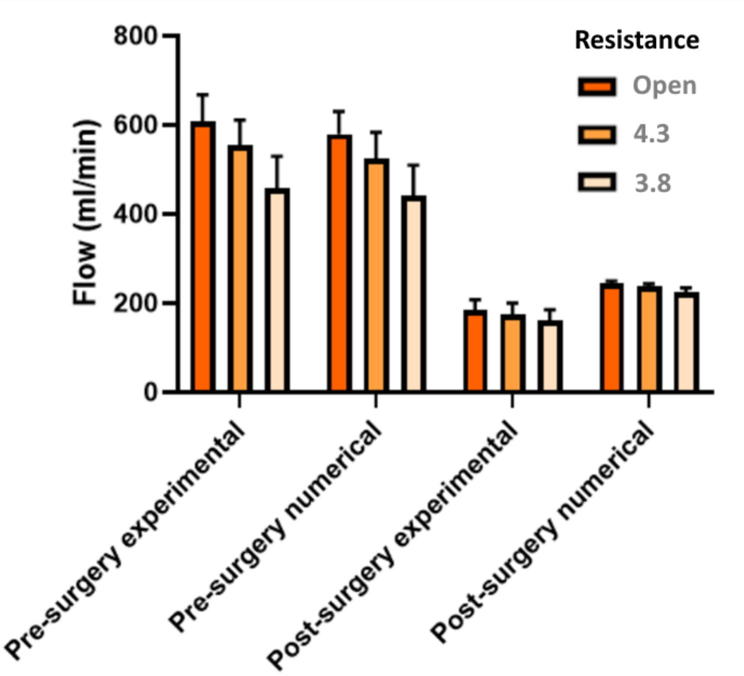

Fig. S3 Flow in experimental and numerical models.

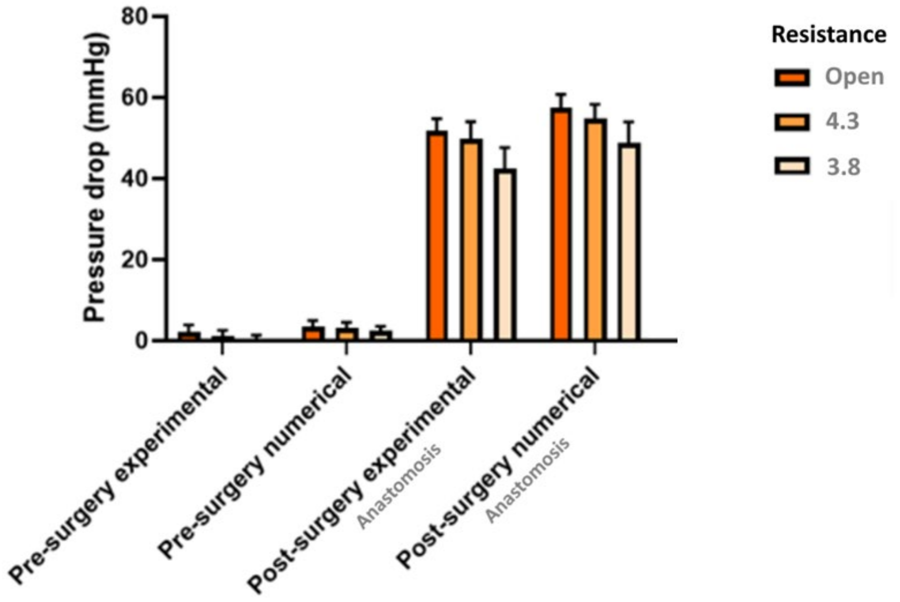

Fig. S4 Pressure drops in experimental and numerical models. The pressure drop over the donor graft approached 0 at all measurements on both the experimental and numerical models.

DISCUSSION

We currently accept the difference in absolute values between the experimental and numerical settings in the post-surgery situation since the absolute deviation was consistent for all measurements. This consistency lead to similar general trends between the experimental setting and the numerical model. These trends showed the highest pressure drop and flow at the lowest resistance (i.e. open), and a decrease in flow and an increase in the pressure drop after application of the ELANA Heart Bypass. No pressure drops were found in the donor graft, probably due to the large diameter (approximately 5 mm) of the urethrae.

Even though there were significant differences between the absolute outcome parameters of the post-surgery experiments and numerical model, these differences were not found for the pre-surgery situation. This indicates that the differences found between the post-surgery mock-loop experiments and numerical model are not due to factors which also apply to the pre-surgery mock-loop and numerical model (e.g. the elasticity of the vessel wall or the input parameters such as the applied resistance). That being said, some physical explanations remain reasonable. Of uttermost importance is, since an ELANA Heart Bypass prototype was used in the experiments, application was not uncomplicated. Whereas the ELANA is supposed to create two exactly opposing arteriotomies in both the donor graft and coronary artery, the prototype ELANA did not create a full arteriotomy in the ureter that mimicked the coronary artery. We therefore had to open the anastomosis and finalize the arteriotomy by hand in most experimental cases. This extra manipulation could very well explain a difference in geometry between the experimental and numerical models. Opening the anastomosis could cause shifting of the vessels and thereby skewed arteriotomies, resulting in a smaller anastomosis. Manipulation on the arteriotomy itself could have caused rupturing of the vessel wall, resulting in a larger arteriotomy. Yet the arteriotomy could also be smaller, in case less vessel wall tissue was removed by hand. Another explanation for the difference in absolute values could be the induction of flow disturbances caused by the curvature and narrow opening of the anastomosis, since the flow within the anastomosis is found at the edge between the laminal and the transitional flow regime.

Since general trends remained consistent between the numerical model and the experiments, we do not expect these factors to influence the current calculations. The deviation; 11% higher pressure drop and 29% higher flow rates for the numerical model, was consistent for all post-surgery measurements. Applying this variance in the final calculations of this study, does not change the observed trends significantly.

For more details on the validation methodology, the results, or the statistics, please contact the corresponding author (h.crielaard@erasmusmc.nl).
